# Improved spatial memory for physical versus virtual navigation

**DOI:** 10.1088/1741-2552/ade6aa

**Published:** 2025-07-11

**Authors:** Shachar Maidenbaum, Vaclav Kremen, Vladimir Sladky, Kai Miller, Jamie Van Gompel, Gregory A Worrell, Joshua Jacobs

**Affiliations:** 1 Department of Biomedical Engineering, Ben-Gurion University, Beer Sheva, Israel; 2 Department of Neurology, Mayo Clinic, Rochester, MN, United States of America; 3 Department of Neurologic Surgery, Mayo Clinic, Rochester, MN, United States of America; 4 Department of Biomedical Engineering, Columbia University, New York, NY, United States of America; 5 Department of Neurological Surgery, Columbia University, New York, NY, United States of America; 6 Faculty of Biomedical Engineering, Czech Technical University in Prague, Kladno, Czech Republic; 7 Czech Institute of Informatics, Robotics, and Cybernetics, Czech Technical University in Prague, Prague, Czech Republic; 8 School of Brain Sciences, Ben-Gurion University, Beer Sheva, Israel

**Keywords:** spatial memory, navigation, physical movement, augmented reality, virtual reality

## Abstract

*Objective*. Virtual reality (VR) has become a key tool for researching spatial memory. Virtual environments offer many advantages for research in terms of logistics, neuroimaging compatibility etc. However, it is well established in animal models that the lack of physical movement in VR impairs some neural representations of space, and this is considered likely to be true in humans as well. Furthermore, it is unclear how big the disruptive effect stationary navigation is—how much does physical movement during encoding and recall affect human spatial memory and representations of space? What effect does the fatigue of actually walking during tasks have on participants—will physical movement decrease performance, or increase perception of difficulty? *Approach*. We utilize Augmented reality (AR) to enable participants to perform a spatial memory task while physically moving in the real world, compared to a matched VR task performed while stationary. Our task was performed by a group of healthy participants, by a group of stationary epilepsy patients, as they represent the population from which invasive human spatial signals are typically collected, and, in a case study, by a mobile epilepsy patient with an investigational chronic neural implant (Medtronic Summit RC + S^TM^) streaming real-time continuous hippocampal local field potential data. *Main results*. Participants showed good performance in both conditions, but reported that the walking condition was significantly easier, more immersive, and more fun than the stationary condition. Importantly, memory performance was significantly better in walking vs. stationary in all groups, including epilepsy patients. We also found evidence for an increase in the amplitude of the theta oscillations associated with movement during the walking condition. *Significance*. Our findings highlight the importance of paradigms that include physical movement and suggest that integrating AR with movement in real environments can lead to improved techniques for spatial memory research.

## 1. Introduction

Where did I leave my keys? Where did I park my car? As we go about our day we constantly perform spatial memory tasks, in which we form and utilize associations between various objects and specific locations. To understand how spatial memory works, one can perform experiments in the real world, by placing items in different locations and asking participants to remember a given object’s location. Such experiments are inherently cumbersome in the real-world—e.g. to test spatial memory for multiple items in different locations one would need to collect those items, manually position them around the environment for each trial, all while being restricted by physical limitations such as the available environment, equipment, and the need to do so consistently without providing improper hints to participants etc. These technical difficulties have led to the popular use of virtual reality (VR) environment based paradigms for studying spatial memory (e.g. for reviews, see [1–3]). VR places the user in a simulated reality which we can control, thus avoiding these limitations. The vast majority of this work currently still utilizes desktop-based environments rather than immersive headsets as these older-style environments are simpler to use and run, do not require dedicated hardware interfaces, and are more compatible with neuroimaging and physiological recording. This use of virtual environments raises an important question—are spatial memory and navigation in virtual environments informative about the same processes as in the physical world?

Desktop based virtual environments lack the physical motion, level of immersion, and idiothetic (internal self-motion) cues of real-world navigation, which may lead to differences in performance (see [4] for a review highlighting these differences). Beyond impairing the environment’s perceived realism, these missing aspects may lead to changes or disruptions in the underlying neural processes and evolutionary evolved mechanisms for spatial memory. Indeed, results from animal models demonstrate that some spatial signals might be disrupted or degraded in virtual environments. For example, Aghajan *et al* [5] found that rodents navigating in VR had disrupted place coding, though see [6] for a counter example. While these challenges are clear, the extent of the actual differences they lead to in terms of spatial memory accuracy in humans is less clear, and thus it is unclear whether stationary, VR navigation platforms serves as an adequate model for natural ambulatory navigational behavior. How important is it for neuroscientists to perform navigation experiments in the real world with actual movement?

**Figure 1.**
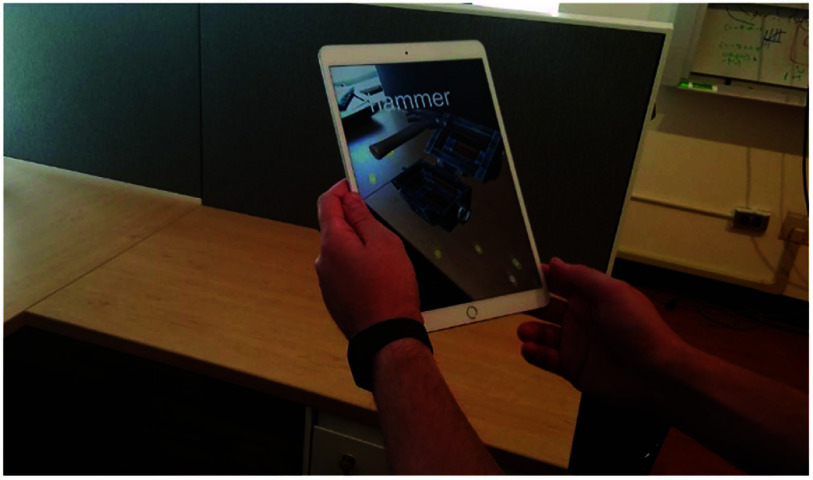
Demonstrating the tablet interface for our ambulatory augmented reality spatial memory task. In this image one can see the tablet held by a user with a target augmented reality chest imposed on the environment, and the empty table on which the chest is superimposed.

Augmented reality (AR) has recently emerged as a powerful new tool to enable spatial-memory paradigms in the real world while including physical movement (e.g. the widespread use of Pokemon Go [7]; see review of this trend in [8]). Unlike VR, in which the user is immersed in a completely separate virtual world where all of the sensory information is virtual, with AR, a user views virtual (or ‘augmented’) objects overlaid on the real world, and this hybrid environment can be viewed via dedicated interfaces such as head mounted displays and smart-glasses, or via commonplace interfaces such as smartphones, and tablets [9]. This enables users to walk around any environment while it is augmented via computational means with targets, landmarks and more. Thus, AR offers a solution for studying spatial memory with the advantages of both real world and virtual paradigms. It allows users to naturally move through their environments, while also providing experimenters precise flexibility and control by having virtual objects and landmarks placed at controlled locations within a real environment with experimentally controlled timing. Previous work on AR and spatial memory is limited—Furio *et al* [10] ran an augmented-reality spatial memory test for children, and showed that it elicits performance patterns that correlate with those seen with more traditional measures. However, this study was limited by the use of fixed physical points for augmented objects via QR codes, and did not compare participant’s performance to a matched virtual version of the same task. Similarly, Khademi *et al* [11] and Mousavi Hondori *et al* [12] used AR for spatial-motor rehabilitation, but did not include a memory element or a direct comparison to VR. Given AR’s potential, we aim here to study empirically whether the ability to walk around in an AR paradigm (figure 1) leads to differences in spatial memory performance compared to a classic stationary VR version of the same paradigm in a matched environment. We examined both memory accuracy as well as participants’ reports of their engagement and enjoyment in the task versions.

We hypothesized that the use of a task with actual walking (our task’s ‘AR condition’) would utilize additional neural systems related to locomotion and internal perception and that this would lead in turn to improved performance and improve the users’ subjective experience. However, we also considered that participants might perform better and experience more enjoyment in the stationary desktop VR condition, as physical walking might lead to increased fatigue that, in turn, could degrade performance.

Although our primary objective here was to compare how spatial memory accuracy shifted between stationary and mobile paradigms, we also tested if patients with chronic epilepsy could perform our ambulatory task, comparing their performance to a larger baseline of epilepsy patients performing the stationary task. This was tested here as chronic epilepsy patients undergoing intracranial electroencephalographic recordings are the rare window into invasive neurophysiology in humans through which many key findings in the realm of spatial neuroscience have been generalized from animal models to humans (e.g. [13] for the first finding of grid cells in humans [14], for finding head direction cells and target location cells, or [15] for the oscillatory correlates of spatial memory and navigation). A common criticism of this body of work is that epilepsy patients may not be representative of the healthy population. Thus, testing that such a task was accessible to them has important ramifications for human neuroscience research and, more broadly, for ensuring that results from research studies generalize to mobile humans.

Finally, we compared navigation-related neural signals between the two task versions via a case-study by enrolling one patient in our task who had an investigational implanted brain-recording streaming device. This novel patient, who was fully mobile with an implanted brain recording device, enabled us to directly test the neural representations in both of the two conditions. Specifically we tested for the existence of a commonly seen specific neural signal related to movement—the hippocampal theta oscillation—which generally increases in amplitude during movement and navigation compared to periods of stillness [16]. Importantly, while previous work has demonstrated this in humans [17], human theta activity is not as clear as that found in animal models, and often appears in lower frequencies. It has been suggested that this may be due to previous work focusing on recordings during stationary tasks, and indeed this difference appeared during movement in a previous ambulatory task [18, 19], but these previous works unfortunately did not include a matched virtual task. Thus, we hypothesized that for both conditions theta-band power should be greater during movement, but that this difference should potentially be more pronounced when physically walking.

## 2. Methods

### 2.1. Paradigm

**Figure 2.**
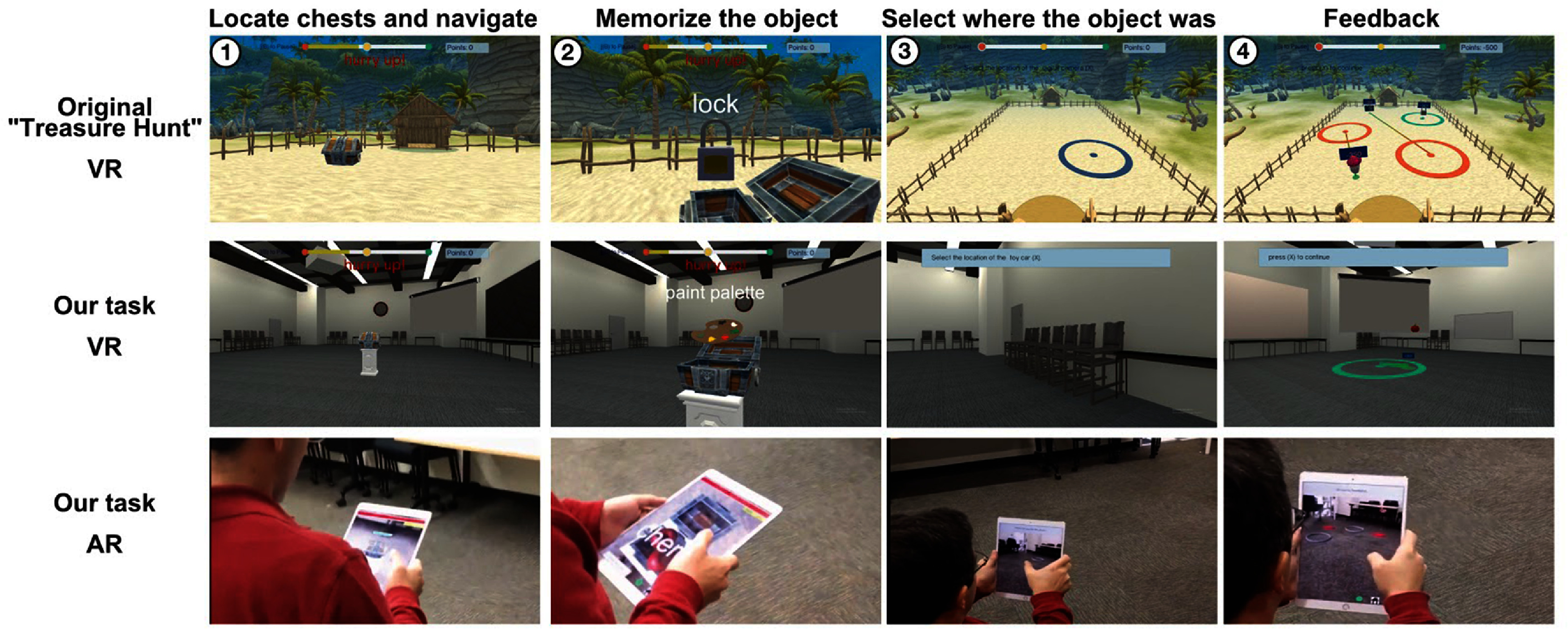
Paradigm. The top row’s screenshots are from the original ‘Treasure Hunt’ task on which we based our paradigm (used in [15]). The middle row is from our adaptation of this task for the one used here in stationary virtual reality, and the bottom row from the mobile Augmented Reality version. In each of these versions, participants perform a series of trials. In each trial they first perform an encoding stage in which they (1) locate 2–4 chests and navigate to them, and then (2) memorize the objects hidden in them. This is followed by a recall stage where participants are cued to (3) mark the location of specific objects and by a (4) feedback stage in which the true location and the participants’ selections are revealed.

We developed matched ambulatory AR and stationary desktop VR versions of the ‘Treasure Hunt’ spatial memory task previously used behaviorally [20] and during stationary neural recordings [21]. Treasure Hunt is an object–location associative memory task in which participants are asked to remember the locations of different hidden objects scattered throughout a virtual environment. Whereas the previous studies had participants perform Treasure Hunt in a tropical beach environment rendered only in VR, here we asked participants to perform the same treasure hunt task in a conference room, matched in both VR and AR implementations (figure 2).

In each trial of Treasure Hunt, participants first perform an ***encoding phase*** (figure 2, Columns 1–2), in which they navigate to a series of treasure chests, each of which is positioned at a random spatial location. When the participant reaches a chest, it opens, revealing an object whose location they are asked to remember. The participant then walks to the next chest. After a series of these learning events, a short ***distractor phase*** begins. Here an animated rabbit runs through the environment, which the participant is instructed to catch. Chasing the animal during the distractor phase serves two purposes, distracting the participant from rehearsing their prior memories and also moving them away from the location of the last remembered object. Next, during the ***retrieval phase*** (figure 2, Column 3), participants are shown the name and image of each object and asked to respond by walking to and indicating the location where that object was encountered. After recalling the locations of all of the trial’s objects, they receive feedback on their response accuracy in the ***feedback phase*** (figure 2, Column 4). Here the participants view every object’s correct location as well as their response location for each object, with lines linking the two. Participants receive points based on their response accuracy and speed in the main spatial-memory task and on their performance on the distractor task. Each trial of Treasure hunt is independent from previous trials as it includes new target objects in new locations.

Participants performed 20 trials of the task in each condition. Each trial included either 2 or 3 target chests and 1–2 empty chests. Thus, each participant viewed a total of ∼50 spatial memory targets for each condition. The overall experiment took participants ∼90–120 min, including time to complete a questionnaire and to walk between the rooms where the stationary and ambulatory conditions took place. Approximately half of the time was spent on the stationary condition and half on the ambulatory condition. Participants used a handheld tablet to view the environment with AR in the ambulatory setting and a standard desktop screen and keyboard for the stationary condition.

### 2.2. Implementation

**The VR task for the stationary condition.** The stationary VR version of Treasure Hunt was developed for Windows using the Unity3D game engine (Unity Systems, USA). We replicated the real-world testing environment used in the mobile AR version of the task, and which had an experiment area size of 7 m × 11 m, using 3D modeling software, such as Blender. During the process of creating a virtual environment that served as a replica of the AR testing environment, we were careful to preserve the dimensions of the room as well as the arrangement of different objects like chairs and tables along the peripheral walls of the environment.

**The AR task for the ambulatory condition.** The AR version of the task was developed for iPad using Unity3D and ARKit (Apple Inc., USA), the latter of which is Apple’s library for allowing development of AR applications for iOS devices [22]. ARKit uses a technique called ‘*visual-inertial odometry’*, which combines motion sensing information from the accelerometer of the iOS device with computer vision analysis of the scene visible to the back-facing camera. It recognizes notable features in the scene image, tracks differences in the positions of those features across video frames, and compares that information with accelerometer data. Using that information, it is able to track the position of the participant holding the iPad accurately within the AR coordinate space. This feature has been tested extensively, and has been shown to be stable over time compared to other methods [23]. Specifically, it has been shown to have a drift of only several centimeters in similar timeframes and environments as our experimental environment. Additionally, note that as every trial was only several minutes long, any potential drift between the encoding and recall stages would be a mere fraction of that and as each trial is independent of the others this fraction is the only drift that might be reflected in the results of each trial. Finally, any significant spike in drift would be noticed in real-time by the participants (e.g. targets appearing outside the experiment area) who did not report such an occurrence.

### 2.3. Healthy participants

Twenty two healthy participants performed the experiment. Due to a technical issue, the logs for the ambulatory condition for four participants were not usable. For these participants, we still used their full stationary results and their questionnaire answers, but note that excluding them does not significantly change any of our results. Power calculations with power = 0.8 and effect size of Cohen’s *D* = 1 on each of the calculations performed below show that our sample size is well powered for each of the analysis performed here. These calculations were performed based on the first 4 participants, and were then verified on the final group.

### 2.4. Ethics

The experiments were approved by Columbia University’s institutional review board (approval AAAR5000) in accordance with the Helsinki declaration, and all participants gave informed consent and were compensated for their time.

### 2.5. Epilepsy patients

We enrolled four additional patients in our study, to verify that patients with chronic epilepsy could indeed perform the ambulatory condition given the importance of this population to spatial neuroscience research. One of these participants was a patient implanted with an investigational deep brain stimulation sensing system, the investigational Medtronic Summit RC + STM. The RC + S is an experimental implantable device for focal epilepsy with sensing and electrical stimulation capabilities. Compared to other available devices, RC + S has the unique advantage streaming continuous local field potential (LFP) to a distributed cloud computing environment that enables tracking electrophysiology and behavior (clinician, researcher) for intelligently adapting brain stimulation. The major advances of the RC + S include uninterrupted iEEG telemetry of multi-node LFP to an epilepsy patient assistant application for data storage, analysis, and cloud computing (see [24–27] for different aspects of the RC + S and it is use clinical and research purposes). In the context of our current study, it enables chronic neural recording as patients are ambulatory, untethered and can freely walk in natural environments. As a case study, a single patient with a chronically implanted RC + S implant took part in our experiment for proof-of-concept of the task, and to enable exploration of neural representations of spatial behavior while walking virtually vs. walking physically. We recorded from four bipolar channels located bilaterally in each hippocampi and anterior thalamus which were localized by the surgeons as part of the implant surgery and was verified via CT and fMRI neuroimaging. Note that the implant can only stream information from four channels at a given time, and we prioritized the hippocampus given it is critical role for spatial memory and navigation. These patients with drug-resistant mesial temporal lobe epilepsy were enrolled under ethical approval (FDA IDE: G180224 and Mayo Clinic IRB: 18-005483) in the mayo clinic.

We compare these patients to a larger dataset of 63 epilepsy patients who performed the stationary version of the task [20].

### 2.6. Statistics

**Performance measures.** Our main measure of performance was how accurately the participant remembered the location of each object. To measure this, we computed the **error distance** between the selected location and the target location—i.e. the raw Euclidean distance between the coordinate of the location the participant selected for their response and the actual target object’s coordinate. We then corrected this distance metric following the procedure described in [15], by comparing it to the distances between 100 000 points randomly generated inside the environment and each target and assigning the percentile as the **corrected error distance**. The corrected error distance is thus the relative rank among these 100 000 distances. The purpose of this corrected error distance measure is that it adjusts for situations in which different target locations can be biased. This can occur, for example, if the target is in the center of a rectangular environment, the maximum error distance is at most half of the diagonal versus a target in the corner of the environment where the maximum distance is the full diagonal length of the rectangular environment. We also extracted the median randomly generated distance as representing the **chance level** for each trial. Beyond these efforts, we note that repeating our analyses with the uncorrected distance errors leads to equivalent results.

**Statistical comparisons of subjective experience.** We first tested the differences in participants’ subjective scoring of difficulty, immersion and enjoyment using a signed-rank test, since these values were discrete and do not distribute normally.

**Statistical comparisons of behavioral performance.** To statistically compare the participants’ memory performance between conditions, we calculated the mean corrected error distance per participant in each condition, and then performed a signed-rank test. To test whether each individual participant performed above chance we used the values described above—the participant’s uncorrected error distance scores and their matching trial-specific chance value generated by taking the distance at 50% in the correction method described in the previous section. We then pairwise tested the relationship between the pairs of selected distances and the surrogate ones using a signed-rank test. Significance levels were corrected via Bonferroni correction for multiple comparisons. These comparisons enable us to directly test in the next section whether participants’ spatial memory performance in each condition was significant and whether physical movement leads to a significant advantage. Finally, we compared the patients’ behavior to a baseline of 63 epilepsy patients who performed the task while stationary using a unpaired rank-sum test.

**Analysis of neural data.** The neural LFP data was extracted from the RC + S implant, from the channels in the patient’s right hippocampus, left hippocampus, right anterior thalamus and let anterior thalamus, sampled at 250 Hz. The behavioral and neural devices were synchronized via joint time setting on the logs of each and verified via a sequences of motions, which were measured on the accelerometers of both the implant and the tablet, enabling a second backup alignment. We then used Matlab to interpolate the behavioral data from 60 Hz to 250 Hz to match the neural data’s sampling rate, and aligned the neural data with the behavioral log to a joint timeseries. We extracted theta power (5–9 Hz) per time point using the Hilbert transform, and then compared mean power during times in which the patient was moving to mean power during times in which the patient was not moving within both the ambulatory and virtual conditions.

## 3. Results

In order to understand spatial memory in the real world, healthy participants performed our version of the ‘Treasure Hunt’ spatial memory task in the ambulatory and virtual conditions. Condition order was randomized between participants to avoid order effects of learning and fatigue. We assessed participant performance by measuring spatial memory accuracy via their performance and user experience via questionnaires. Participants also filled out a standard questionnaire that assessed their spatial abilities (The Santa Barbara Sense of Direction scale, SBSoD [28]).

All participants were able to successfully complete the experiment, which included physically walking ∼1 km within the experiment room during the ambulatory condition.

**Performance in each condition separately.** We first tested whether participants could perform the task well by assessing whether their performance was above chance. In the walking condition the corrected error distance across participants was 0.08 ± 0.01 (normalized units, 0 representing best memory, 0.5 chance, and 1 representing worst memory), and all individual participants corrected error scores were significantly lower than 0.5 as required. Indeed, when comparing each participant’s raw error distances to their own corresponding chance levels (the value ranked at 0.5 from the surrogate distribution) we found that all of the participants were able to perform the task significantly above chance (all p’*s* <10^−7^). This demonstrated that participants were consistently able to respond at locations relatively close to the actual memory target position.

Next, we measured performance while participants performed the task in the matched stationary condition. We found that here too participants were able to perform the task significantly above chance (corrected error distance = 0.16 ± 0.01, all p’*s* <0.03). We also compared their performance to a wider baseline of data from healthy participants in the standard implementation of the ‘Treasure Hunt’ task from [20] and found that the results were in line with this baseline (*p* = 0.65, unpaired two-tailed *t*-test). This demonstrates the validity and fit of our task for successful testing spatial memory despite the use of a different virtual environment compared to the earlier studies (as here we used the matched room rather than the beach used in previous work [15]).

**Figure 3.**
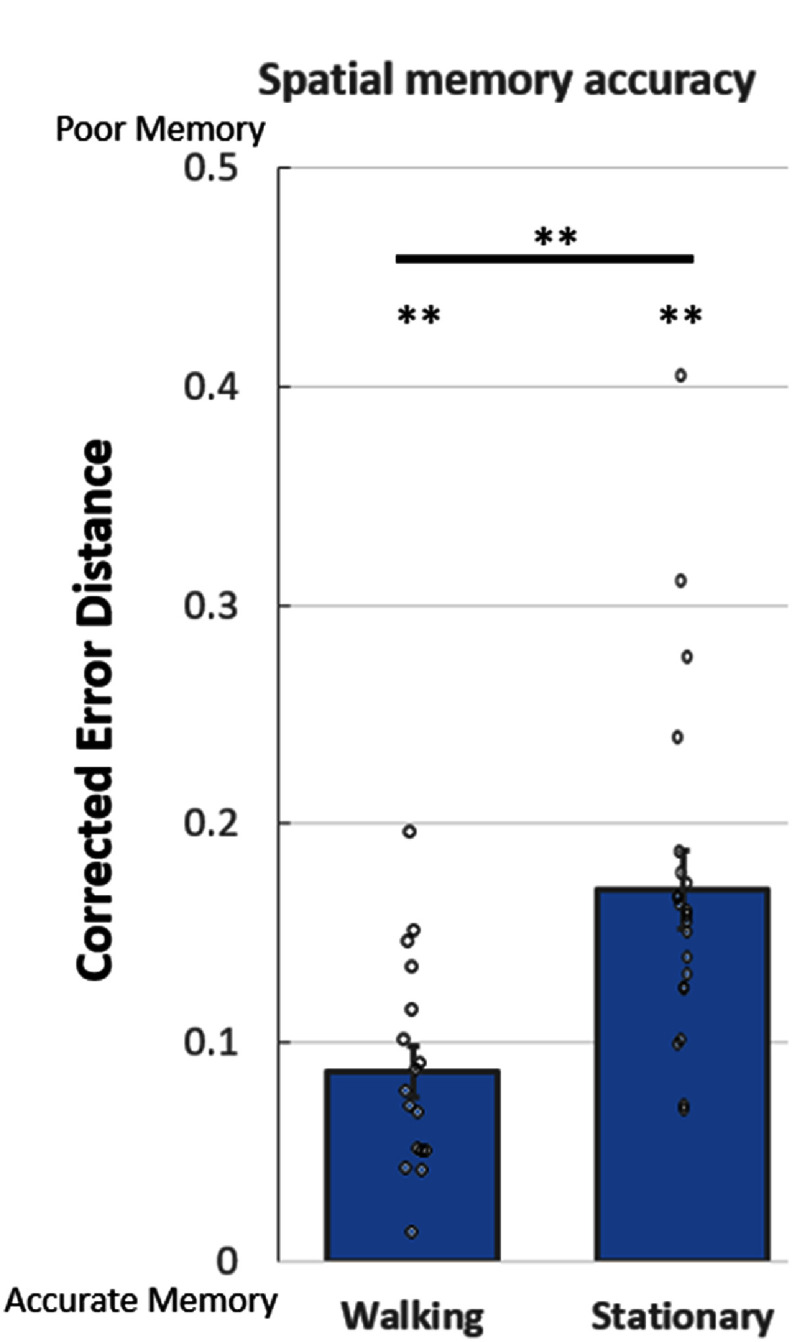
Spatial Memory accuracy. Corrected error distance scores reveal that participants showed significant spatial memory in both conditions compared to chance level established by the surrogate distributions. When comparing physically walking to stationary virtual walking, we found a significant advantage for physical movement. Comparison between conditions was performed with the Signed-Rank test. Comparison of each bar to chance was performed by the procedure described above. For both tests ** indicate *p* < 0.01 corrected.

**Comparing physically walking and stationary virtual walking.** We next compared the performance of participants between the ambulatory versus stationary conditions. Here we found that participants were significantly more accurate when physically walking than when stationary with virtual walking (corrected error distance was 0.08 ± 0.01 and 0.16 ± 0.01 respectively, Cohen’s *D* = 1.49, *p* < 0.001, rank-sum test) (figure 3)—performance when physically walking was twice as accurate!

At the single participant level, only three participants (15%) showed better accuracy in the stationary conditions, while all others (85%) had higher accuracy in the ambulatory condition showing that this effect was not just driven by a small number of participants (binomial test, *p* < 0.002).

In order to give an intuition for the meaning of this corrected error size, we looked also at the uncorrected results. These translate to a raw mean distance error on the order of 2.19 m in the real world for the stationary condition (VR) and 1.18 m for the ambulatory condition (AR). This gap matches the corrected results indicating that participants were about twice as accurate. Note that this difference is in the order of magnitude of a meter, while the potential drift and inaccuracies in the AR system used are in the order of centimeters, precluding any such potential errors from having a significant effect on our results.

We tested our results for order effects across conditions and did not find any such effects. Participants who performed the walking condition first did not perform at a different level from those who walked second (*p* = 0.92, rank-sum test on participants mean corrected errors in the ambulatory condition between participants who performed it first and those who performed it second) and stationary first was not significantly different from stationary second (*p* = 0.75, rank-sum test on participants mean corrected errors in the stationary condition between participants who performed it first and those who performed it second).

**Figure 4.**
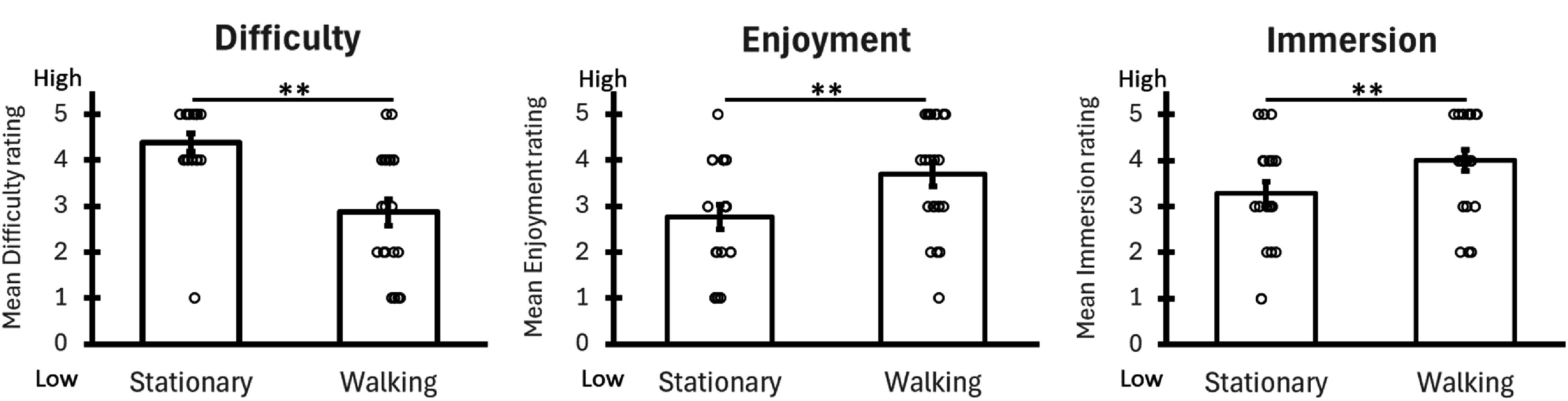
Subjective experience of walking physically vs. virtually. Participants subjectively reported that the physical walking condition was significantly easier, more fun and more immersive than the virtual walking condition. Comparison between conditions was performed with the Signed-Rank test. ** indicate *p* < 0.01 corrected.

To better understand the source of this improved performance during the ambulatory condition, we compared participants’ subjective experiences between the two conditions (figure 4). Participants subjectively reported that the ambulatory version was easier than the stationary condition (Likert scale 1-5, 1 easy and 5 difficult; Means = 2.9 ± 1.3, 4.4 ± 0.5 respectively, *p* < 0.01), more enjoyable (Likert scale 1–5, 1 fun and 5 not fun; Means = 3.6 ± 1.1, 2.7 ± 1.1 respectively, *p* < 0.01) and more immersive (Likert scale 1–5, 1 low immersion and five high immersion; Means = 3.9 ± 0.9, 3.2 ± 1.1 respectively, *p* < 0.01). These ratings matched participants’ comments: ‘Overall, the mobile AR was fun and immersive’ S3 ‘When I feel disconnected from my body, I had difficulty to estimate my location accurately. “ S16 ‘to sense the space in VR is much harder.’ S22. Note that this preference for the walking condition persisted despite participants in the ambulatory needing to physically walk for ∼20 min, covering over a kilometer of real world distance. These factors suggest that fatigue was not a serious constraint in our task.

**Spatial memory performance and sense of direction.** We used the SBSoD scale questionnaire to assess how each participant perceives their own spatial abilities [28]. This measure has been shown to strongly correlate with many other spatial measures (e.g. perspective taking abilities [29], the big-5 personality traits [30], and driving space [31]). Therefore, we correlated participants’ scores on the Santa Barbara questionnaire with their performance in each of the conditions. We did not find a correlation between SBSoD to performance in the stationary virtual walking condition (*r* = 0.06, *p* = 0.79), but did find a positive correlation with performance for the physical walking condition at a trend towards significance (*r* = 0.4, *p* = 0.08). This result was also consistent with participant’s subjective responses. Several participants reported that physical walking felt closer to natural behavior. For example: ‘I felt like I was doing something totally different when actually walking, this just felt natural’ (S62), ‘In VR I felt like my body was not connected to my movements and I was totally disconnected’ (S45).

**Figure 5.**
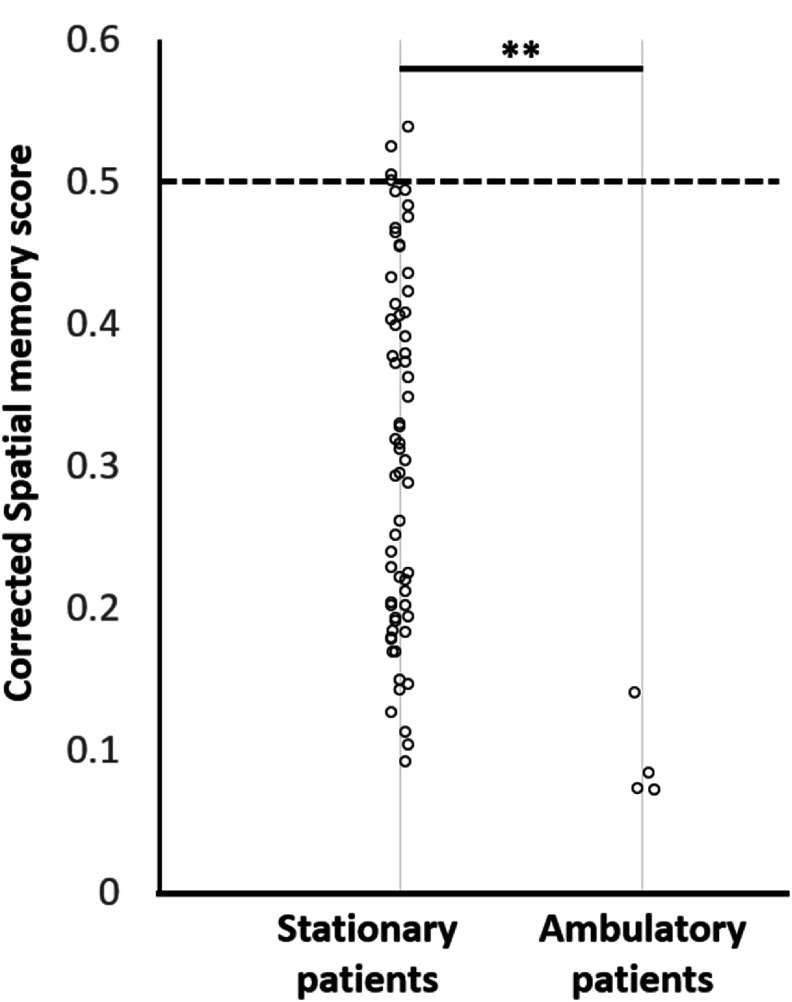
Epilepsy patients sitting vs. walking. We compared our 4 epilepsy patients who performed the walking condition (right) to a baseline of 63 epilepsy patients who performed the standard sitting condition and found a significant advantage for walking (rank-sum, *p* < 0.001). ** indicates *p* < 0.01 corrected.

**Extending results to ambulatory epilepsy patients.** Would these results extend also to patients with epilepsy, which are a critical population for researching spatial neuroscience questions? To test this, we recruited a set of four epilepsy patients as a case study to test if their performance when walking would match the performance distribution of walking or of the stationary conditions. We found that indeed, the four patients were able to perform the task significantly above chance (all *p* ≪ 0.01), and further that their distribution and performance levels matched that of the ambulatory condition in healthy participants (*p* = 0.68 when compared to ambulatory healthy, *p* < 0.03 significantly better than healthy participants in the stationary condition). Comparing their scores to data from patient’s performing the static version of the original Treasure Hunt task [20] showed that these patients exhibited significantly better performance (rank-sum, 0 < 0.001) (figure 5).

**Neural representation of physical movement.** We then asked how moving physically would affect the underlying neural hippocampus LFP signature for movement. We examined this issue with recordings from a single case study patient who was implanted with a Medtronic RC + S with streaming hippocampus LFP data. Given our literature based prediction above we focused only on the theta band.

**Figure 6.**
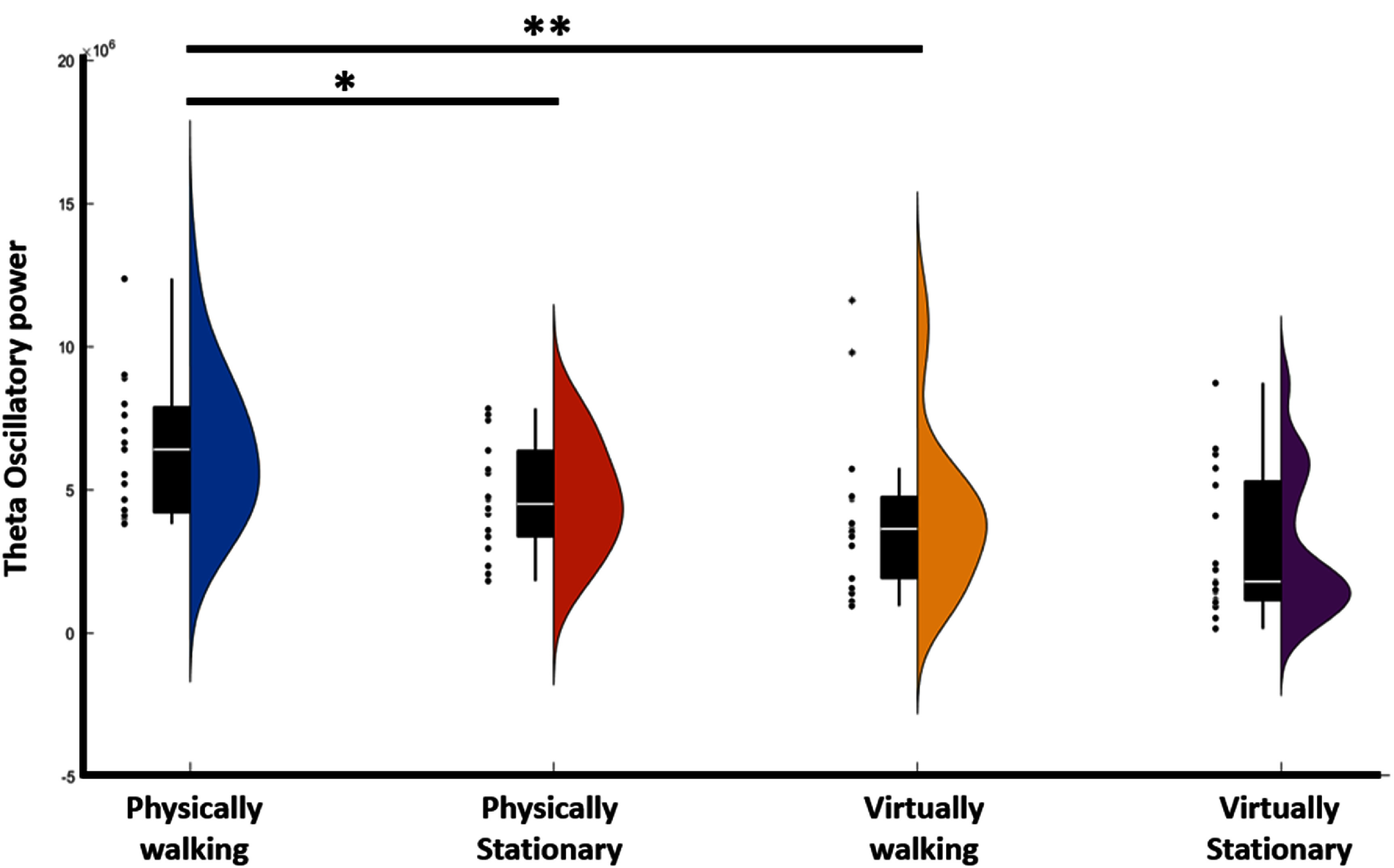
Theta oscillations in stationary vs. physically moving. Participants displayed increased theta for moving > standing in the physical conditions, and the physical walking showed higher theta than virtual walking. An ANOVA revealed a significant difference across conditions. Comparison between conditions was performed with post-hoc tests on power values on the mean values per trial for each condition. ** indicate *p* < 0.01 corrected, * indicates *p* < 0.05 corrected.

An ANOVA on trial level results indicated an effect of condition on theta power (F(3,54) = 6.1, *p*< 0.005) (figure 6). Consistent with our predictions, post-hoc analysis revealed that there was greater theta power (5–9 Hz) in the left hippocampus channel during movement in the physical walking condition (Physical Walking vs standing *p* < 0.05). Movement while ambulatory elicited significantly more theta power theta moving virtually while stationary (Physical Walking vs Virtual walking *p* < 0.01). The right hippocampus showed a similar pattern but did not reach significance. As our hypothesis was based on a signal found in the hippocampus of animal models we did not expect to find it in the two anterior thalamic channels, and it indeed did not appear in them. These results emphasize the importance of physical movement and the potential for AR-based mobile tasks, with real physical rather than virtual movement, to more strongly engage the hippocampal network as indexed via theta rhythms.

## 4. Discussion and future work

Our main finding was that when comparing spatial memory directly between performing a spatial memory task while physically walking with an AR interface to performing a matched stationary task with virtual walking on a computer screen, accuracy was twice as good in the condition which included physical walking. Further, participants found the ambulatory version of the task to be significantly easier, more fun, and more immersive. We also found preliminary evidence that this is true not only for spatial memory accuracy but potentially also for the underlying neural signals, extending the work of [18, 19].

These results also suggest that AR-based navigation tasks have potential for improving our ability to probe human spatial behavior and the underlying neuroscience. Our results show nearly a doubling of spatial accuracy when participants can physically move, indicating that a critical component is missing in stationary tasks and that there is a need for further naturalistic spatial navigation research in which participants can physically move.

**The effect of moving on behavior.** While we expected improved performance in our task, we also expected this difference to be relatively modest and tempered by effects of fatigue. Instead, our results show a highly significant difference of doubling the accuracy when physically moving. This suggests a gap that needs to be considered carefully in work which relies solely on virtual spatial memory.

**Why is performance better when physically moving?** As suggested in the introduction, one aspect may be the lack of many physiological cues while navigating virtually. These include sensory data from the proprioceptive and vestibular systems, information from motor afferents etc. This missing information might in turn lead to impairments in the neural representation of the environment during encoding on the one hand, and impairments in neural location updating and calculation during recall. These impairments could lead to weaker signals and weaker recruitment of the brain’s navigation network, or alternatively lead to degraded and possibly even erroneous representations. Given that fMRI does not enable participants to move, and that EEG tends to accrue significant artifacts while moving and does not readily offer access to key deep brain regions involved in spatial memory (e.g. hippocampus, entorhinal cortex) testing this question directly is difficult. We attempted to test it via a case study with invasive recordings, and our current findings indeed supported this direction.

**Reality modality.** Although our results are suggestive of differences between the walking and stationary conditions, it must be acknowledged that there is a second parameter differing across the conditions—the reality modality. Specifically, the ambulatory condition utilized AR in the real world, while the stationary condition utilized a fully virtual environment. To our knowledge, spatial memory performance has not been directly compared between mobile AR and mobile VR using matched tasks. However, the relationship between AR and VR has been explored for many other realms, with emphasis on education and training. These include testing educational applications, such as teaching about recycling [32], the water cycle [10], multiculturalism [33], forensic medicine [34] and English as a second language [35]. These studies all found that the use of AR was at least equivalent to VR for the tested tasks.

While AR and VR have not been directly compared for spatial memory, two other types of spatial memory comparisons involving VR are relevant to our question. First, performance in VR has been compared extensively to real-world performance, demonstrating the potential for similar levels of accuracy and for transfer between training in one to the other—but also the limitations and gaps that remain [36, 37].

From a neuroscience perspective, the extent to which the neural signals underlying behavioral performance are similar between virtual and real-world environments is currently debated, with some studies showing that signals are maintained while others finding significant differences (see an example of such conflicting results in [5, 38] and examples of reviews with conflicting approaches in [4, 39]). The case study here supports the importance of physical movement for strengthening these representations. Furthermore, in recent years immersive VR setups that enable physical movement have become available. These include HMD setups with either natural walking in small safe environments, or on an omni-directional treadmill or simply stationary while allowing naturalistic head movements. The use of these setups has been compared to traditional screen-based desktop VR, showing general equivalence between the methods, with various advantages to walking over stationary conditions [40–42]. From the neuroscience perspective, it has been suggested based on animal studies that naturalistic head movements may be sufficient to elicit neural spatial signals in VR that might be missing in fully virtual paradigms [6]. Note however that these types of walking with an HMD, especially on a treadmill, may still hold considerable subjective differences to realistic real-world walking (e.g. [40, 43, 44]) and thus if performance is indeed improved by more natural physical walking then we would expect performance in ambulatory AR to be between performance in immersive VR to performance in real-world paradigms.

Furthermore, while AR has the advantages of both real world and immersive VR, it is still in its current technological level a compromise between them. Although AR provides much more flexibility than regular real-world environments, it still does not match the flexibility of fully immersive VR as it continues to be limited by the underlying layout of the actual physical environment. The naturalistic feeling from using AR tends to break down in complex environments with occluding surfaces where sometimes the accuracy of the positioning of augmented objects can be problematic, which led to our choice of an open arena paradigm here to avoid such issues. In both of these cases, further advances in AR technology will continue to mitigate these differences to a great extent across a wide range of fields—for general computing [45], for psychological research [46], for rehabilitation [47], for education [48] and for clinical use [49]). Thus, though AR tools are still new and evolving and we can expect improved results going forward, even current versions can already be utilized to create experiments that are more naturalistic and better capture human performance.

Accordingly, future work should directly test each of these factors, extending our work by performing a task similar to ours in matched environments between stationary VR, mobile VR and mobile AR to disentangle the relative effect of physical motion vs. reality modality.

**Other limitations.** Beyond the challenge of disentangling physical movement from reality modality there could be additional confounding factors inherent to the real world such as potential effects of the real-world environment—e.g. subtle differences that were not modeled virtually or aspects that were perceived in the real world beyond vision such as the feeling of texture on the carpet, breeze from an air conditioner etc. Another category of potential differences are differences in interface, such as walking to a location and then clicking on a tablet screen vs. virtually moving and pressing a key may also play a part. While we hypothesize that their relative influence is smaller than physical motion, these alternative explanations challenge our findings and accordingly they need to be isolated and tested in dedicated experiments. Such an experiment might utilize ambulatory VR tools as described in the previous section to perform the experiment in the same environment, using the same interface and while walking in both conditions and then varying each time only one of these three parameters to determine its effect relative to the rest.

An additional limitation is the limited number of patients, and especially our single participant case study, which limit us to being able to point at the potential of our approach but prevent statistical validity for generalization.

**Potential for Neuroscience Research.** Following earlier advances, AR has the potential of being an extremely powerful tool for psychological and neuroscience research. An important first step, which we contribute to here, is in establishing clear behavioral baselines for performance in AR, to enable better extrapolation and generalization from the much larger existing VR research. Specifically, for the research of spatial memory, one can use current AR tools to test a range of questions in spatial memory research. For example, will we see differences between familiar and unfamiliar environments in the real world? How does memory performance in AR environments vary indoors versus outdoors? The greater ecological validity of AR can offer especially strong potential when combined with mobile neuroimaging (e.g. mobile fNIRS, mobile EEG) and invasive brain recording (e.g. the chronically implanted Neuropace [50] or the RC + S devices as used here [24]). This can enable us to create flexible, but highly controlled, paradigms in naturalistic real world settings, which might allow us to identify novel brain signals that have been previously missing from findings obtained from VR-based paradigms [5]. For this reason we focused here on tablet based AR rather than on head mounted displays or smart glasses, as this avoids clashes between the AR and neuroimaging equipment.

**Potential for rehabilitation.** In addition to basic research, our findings of improved realism and enjoyment for AR-based walking paradigms suggest a potentially useful route for creating translational tools for uses such as rehabilitation. Current research approaches for spatial memory rehabilitation face similar kinds of challenges as spatial memory research, although often the magnitude of these problems is magnified by the need for the paradigms to be accessible to participants with memory impairments [3, 47]. Existing real-world navigation paradigms are often too cumbersome to run in the clinic, not to mention home, and virtual paradigms have not been successfully adopted (e.g. placing multiple obstacles in changing locations for a patient to walk around as they walk up and down a corridor). Existing VR tools on the other hand also suffer from challenges such as the disconnect between patients and their environment, including the clinical staff or helping family members, and the complexity of interfaces. Because AR connects the patient more with their physical surroundings and may be more convenient, intuitive, and enjoyable for individuals with spatial memory impairments, these methods may have special utility for working with these challenged patients—this is a view that has also been previously suggested by others for other rehabilitation realms (e.g. for mental disorders [46, 47], post stroke [51] etc.; broadly reviewed in [52]). Furthermore, beyond the advantages mentioned above regarding naturalness and flexibility, our findings show also that AR has the advantage of being easier to use. For all of these reasons, we see great potential in future use of AR for spatial memory rehabilitation and training, and more generally for the realm of rehabilitation in general.

## 5. Conclusion

Our main impact and novelty are in providing a quantitative measurement of the improvement in spatial memory accuracy that results from walking in the real world with AR compared to a matched stationary task with VR. Our finding that spatial memory encoding while physically walking was significantly easier, more immersive and more fun as compared to virtual walking, and most importantly that performance was significantly more accurate, demonstrates the importance of physical movement for spatial research and the potential of AR tools for spatial memory research and rehabilitation. Our patients demonstrate the potential for use of such systems with clinical populations as well, and specifically with epilepsy patients which are a key target population for invasive neurophysiological recordings in the field of spatial neuroscience. Our case study demonstrates that beyond behavior these effects may extend also to the underlying neural representations challenging us to integrate physical movement into neural experiments as well. These findings hold significant potential as a foundation for future spatial memory research and rehabilitation.

## Data Availability

The data that support the findings of this study are openly available at the following URL/DOI: https://osf.io/preprints/psyarxiv/d5fra. Data will be available from 01 October 2025.
